# Rare key functional domain missense substitutions in *MRE11A*, *RAD50*, and *NBN* contribute to breast cancer susceptibility: results from a Breast Cancer Family Registry case-control mutation-screening study

**DOI:** 10.1186/bcr3669

**Published:** 2014-06-03

**Authors:** Francesca Damiola, Maroulio Pertesi, Javier Oliver, Florence Le Calvez-Kelm, Catherine Voegele, Erin L Young, Nivonirina Robinot, Nathalie Forey, Geoffroy Durand, Maxime P Vallée, Kayoko Tao, Terrell C Roane, Gareth J Williams, John L Hopper, Melissa C Southey, Irene L Andrulis, Esther M John, David E Goldgar, Fabienne Lesueur, Sean V Tavtigian

**Affiliations:** 1 Genetic Cancer Susceptibility group, International Agency for Research on Cancer, 150 cours Albert Thomas, Lyon 69372, France; 2 Genetic of Breast Cancer group, Cancer Research Center of Lyon, Centre Léon Bérard, 28 rue Laennec, Lyon 69008, France; 3 Department of Oncological Sciences, Huntsman Cancer Institute, University of Utah School of Medicine, 2000 Circle of Hope, Salt Lake City, UT 84112, USA; 4 University of Texas at Austin, Austin, TX 78712, USA; 5 Life Sciences Division, Lawrence Berkeley National Laboratory, 1 Cyclotron Road, Berkeley, CA 94720, USA; 6 Centre for Epidemiology and Biostatistics, School of Population and Global Health, The University of Melbourne, 207 Bouverie Street, Melbourne, VIC 3010, Australia; 7 Genetic Epidemiology Laboratory, The University of Melbourne, 207 Bouverie Street, Melbourne, VIC 3010, Australia; 8 Lunenfeld-Tanenbaum Research Institute, Mount Sinai Hospital, Department of Molecular Genetics, University of Toronto, 600 University Avenue, Toronto, ON M5G 1X5, Canada; 9 Cancer Prevention Institute of California, 2201 Walnut Avenue, Fremont, CA 94538, USA; 10 Department of Dermatology, Huntsman Cancer Institute, University of Utah School of Medicine, 2000 Circle of Hope, Salt Lake City, UT 84112, USA; 11 Genetic Epidemiology of Cancer team, Inserm, U900, Institut Curie, Mines ParisTech, 26 rue d’Ulm, Paris 75248, France; 12 Stanford University School of Medicine and Stanford Cancer Institute, 875 Blake Wilbur Drive, Stanford, CA 94305, USA; 13 Department of Epidemiology (Genome Epidemiology Lab), Seoul National University School of Public Health, 599 Gwanak-ro Granak-gu, Seoul 151-742, Korea

## Abstract

**Introduction:**

The MRE11A-RAD50-Nibrin (MRN) complex plays several critical roles related to repair of DNA double-strand breaks. Inherited mutations in the three components predispose to genetic instability disorders and the MRN genes have been implicated in breast cancer susceptibility, but the underlying data are not entirely convincing. Here, we address two related questions: (1) are some rare MRN variants intermediate-risk breast cancer susceptibility alleles, and if so (2) do the MRN genes follow a *BRCA1*/*BRCA2* pattern wherein most susceptibility alleles are protein-truncating variants, or do they follow an *ATM*/*CHEK2* pattern wherein half or more of the susceptibility alleles are missense substitutions?

**Methods:**

Using high-resolution melt curve analysis followed by Sanger sequencing, we mutation screened the coding exons and proximal splice junction regions of the MRN genes in 1,313 early-onset breast cancer cases and 1,123 population controls. Rare variants in the three genes were pooled using bioinformatics methods similar to those previously applied to *ATM*, *BRCA1*, *BRCA2*, and *CHEK2*, and then assessed by logistic regression.

**Results:**

Re-analysis of our *ATM, BRCA1*, and *BRCA2* mutation screening data revealed that these genes do not harbor pathogenic alleles (other than modest-risk SNPs) with minor allele frequencies >0.1% in Caucasian Americans, African Americans, or East Asians. Limiting our MRN analyses to variants with allele frequencies of <0.1% and combining protein-truncating variants, likely spliceogenic variants, and key functional domain rare missense substitutions, we found significant evidence that the MRN genes are indeed intermediate-risk breast cancer susceptibility genes (odds ratio (OR) = 2.88, *P* = 0.0090). Key domain missense substitutions were more frequent than the truncating variants (24 versus 12 observations) and conferred a slightly higher OR (3.07 versus 2.61) with a lower *P* value (0.029 versus 0.14).

**Conclusions:**

These data establish that *MRE11A*, *RAD50*, and *NBN* are intermediate-risk breast cancer susceptibility genes. Like *ATM* and *CHEK2*, their spectrum of pathogenic variants includes a relatively high proportion of missense substitutions. However, the data neither establish whether variants in each of the three genes are best evaluated under the same analysis model nor achieve clinically actionable classification of individual variants observed in this study.

## Introduction

Based on risk and frequency, three classes of breast cancer susceptibility genes or loci are currently recognized: high-risk genes such as *BRCA1* and *BRCA2* (Mendelian Inheritance in Man numbers (MIMs) 113705 and 600185) in which protein-truncating mutations and severely dysfunctional missense substitutions confer a five- to ten-fold increased risk and for which the summed allele frequency in the general population is <1%; intermediate-risk genes such at *ATM* and *CHEK2* (MIMs 208900 and 604373) in which protein-truncating mutations and severely dysfunctional missense substitutions confer a two- to five-fold increased risk and for which the summed allele frequency in the general population may approach 1%; and common, modest-risk single nucleotide polymorphisms (SNPs) which individually have much higher frequency but only rarely confer risk greater than 1.25-fold [1,2]. Linkage analysis provided an effective genome-wide approach for locating high-risk susceptibility genes, and genome-wide association study provided an effective approach for finding risk-associated SNPs. While exome sequencing-based strategies may eventually provide a hypothesis-free, genome-wide approach for identification of intermediate-risk genes, much of our current knowledge base has flowed from candidate gene studies [3].

The MRN complex, formed from dimers of the proteins encoded by *MRE11A*, *RAD50*, and *NBN* (MIMs 600814, 604040, and 602667), plays key roles in DNA double-strand break (DSB) repair, meiotic recombination, cell cycle checkpoints, and maintenance of telomeres [4]. In mice, homozygous knockouts of these genes are lethal [5-7]. Humans born with biallelic mutations in any one of the three genes share a cellular phenotype that includes sensitivity to ionizing radiation, a deficit in DNA DSB repair, and chromosomal instability (MIM 604391; MIM 251260) [8]. Moreover, these people are at risk of severe cancer susceptibility phenotypes. For example, two brothers who were compound heterozygotes for mutations that fell in the amino region of the MRE11A protein died of pulmonary adenocarcinoma before age 20 [9,10], which would seem very unlikely in the absence of an underlying cancer predisposition. Susceptibility to lymphoma is a prominent feature of Nijmegen breakage syndrome, which is caused by biallelic mutation of *NBN*[11]. While too few human biallelic *RAD50* mutation carriers have been identified to reach a conclusion about their cancer susceptibility, more than 20% of mice homozygous for a hypomorphic Rad50 allele (Rad50 p.Lys22Met) that lived past age four months died with lymphoma or leukemia [12].

Breast cancer risks for heterozygous carriers of MRN gene mutations were summarized briefly by Hollestelle *et al*. [3]. Of the three genes, *NBN* has the strongest evidence in support of acting as an intermediate-risk breast cancer gene. This is largely because the truncating variant *NBN* c.657del5 has a high enough frequency among individuals of Slavic origin to be evaluated by case-control analysis, and meta-analysis of nine such studies revealed a combined odds ratio (OR) of 2.63 (95% confidence interval (CI) 1.76 to 3.93) for this variant [13]. Most evidence in favor of *RAD50* rested on a truncating variant *RAD50* c.687delT which has been observed in Finnish cases and controls [14,15]; while subsequent studies in the same and other populations are consistent with the hypothesis that *RAD50* is an intermediate-risk breast or pancreatic cancer susceptibility gene, they have not provided significant supporting evidence [16,17]. Evidence for *MRE11A* rests primarily on the observation of two mutations in the gene from a series of eight non-*BRCA1*/*2* breast cancer families with tumors that showed loss of all three MRN proteins [18].

Previously, we performed case-control mutation screening studies of *ATM*, *CHEK2*, *XRCC2*, and *RAD51* to clarify our understanding of their role in breast cancer susceptibility [19-22]. A common thread across these studies has been use of bioinformatic and statistical approaches designed to detect evidence of pathogenicity from both truncating and splice junction variants (T + SJV) and/or rare missense substitutions (rMS). Here, we apply a case-control mutation screening strategy in an ethnically diverse series of subjects to evaluate *MRE11A*, *RAD50*, and *NBN*. Given that the three proteins form an evolutionarily conserved complex involved in maintenance of genomic integrity, we decided to evaluate the three genes as a single large candidate intermediate-risk breast cancer susceptibility gene with a concatenated open reading frame of 2,774 amino acids - which nevertheless is not quite as large as the 3,056 amino acid open reading frame of *ATM*. Our analysis addresses two related questions: (1) are some rare MRN variants intermediate-risk breast cancer susceptibility alleles, and if so (2) do the MRN genes follow a *BRCA1*/*2* pattern wherein most susceptibility alleles are protein-truncating variants, or do they follow an *ATM*/*CHEK2* pattern wherein half or more of the susceptibility alleles are missense substitutions?

## Methods

### Study sample

The design for this study has been described in detail previously [19,20,22]. Briefly, eligible participants included women ascertained by population-based sampling by the Australian, Northern California, and Ontario sites of the Breast Cancer Family Registry (BCFR) [23]. Subjects were recruited between 1995 and 2005. Selection criteria for cases (N = 1,313) were diagnosis of breast cancer at or before age 45 years and self-reported race/ethnicity plus grandparents’ country of origin information consistent with Caucasian, East Asian, Hispanic/Latino, or African American racial/ethnic heritage. The controls (N = 1,123) were frequency matched to the cases within each center on racial/ethnic group, with age at selection not more than ± 10 years from the age range at diagnosis of the cases gathered from the same center. Because of the shortage of available controls in some racial/ethnic and age groups, the frequency matching was not one-to-one in all subgroups.

Recruitment and genetic studies were approved by the Ethics Committee of the International Agency for Research on Cancer (IARC), the University of Utah Institutional Review Board (IRB), and the local IRBs of the BCFR centers from which we received samples. These local IRBs were the Health Sciences Human Research Ethics Subcommittee of the University of Melbourne, Australia; the Institutional Review Board of the Cancer Prevention Institute of California; and the Research Ethics Boards of Mount Sinai Hospital and the University Health Network, Ontario, Canada. Written informed consent was obtained from each participant.

### Mutation screening

For mutation screening of the coding exons and proximal splice junction regions of *MRE11A* (NM_005591.3), *RAD50* (NM_005732.3) and *NBN* (NM_002485.4), we used 30 ng of whole-genome amplified (WGA) DNA obtained by mixing 15 ng of amplified DNA from each of two independent WGA reactions. The laboratory process was as described in detail for our recent studies of *ATM*, *CHEK2*, *XRCC2*, and *RAD51*[19-22]. Our semi-automated approach, handled by a Laboratory Information Management System (LIMS) [24,25], relies on mutation scanning by high-resolution melt curve (HRM) analysis followed by direct Sanger sequencing of the individual samples for which an aberrant melting curve profile is indicative of the presence of a sequence variant. In a previous work, we showed that the HRM technique showed high sensitivity and specificity (1.0, and 0.8, respectively, for amplicons of <400 bp) for mutation screening by comparing the results with those obtained with Sanger sequencing [26].

For *MRN* amplicons harboring a SNP(s) with frequency ≥1% in either dbSNP or initial amplicon testing, we applied a simultaneous mutation scanning and genotyping approach using HRM analysis to improve the sensitivity and the efficiency of the mutation screening, as described previously [24,25].

All exonic sequence variants, plus intronic sequence variants that fell within 20 bp of a splice acceptor or eight bp of a splice donor, and were either unreported or had an allele frequency of <1% in the large scale reference groups ‘Caucasian Americans’, ‘African Americans’ and ‘East Asians’ based on exome variant server (EVS) and 1,000 genomes project (1000G) data [27,28], were confirmed either by independent re-amplification and sequencing from each of the two independent WGA reaction products and concordant variant calls, or, for five variants, by re-amplification and sequencing from genomic DNA.

All samples that failed either at the primary PCR, secondary PCR, or sequencing reaction stage were re-amplified from WGA DNAs or genomic DNAs. Samples that still did not provide satisfactory mutation screening results for at least 80% of the concatenated MRN coding sequence were excluded from further analysis. Primer and probe sequences are available from the authors upon request.

### Alignments and scoring of missense substitutions

We used M-Coffee [29], which is part of the Tree-based consistency objective function for alignment evaluation (T-Coffee) software suite of alignment tools [30] to prepare a protein multiple sequence alignment for each of the three MRN proteins (MRE11A, RAD50 and NBN) in order to predict the effect of missense substitutions on the proteins and on the activity of the MRN complex. Each alignment consisted of sequences from 16 species: *Homo sapiens*, *Macaca mulatta*, *Callithrix jacchus*, *Mus musculus*, either *Sus scrofa* or *Bos Taurus, Loxodonta africana*, *Dasypus novemcinctus*, *Monodelphis domestica*, *Ornithorhynchus anatinus*, *Gallus gallus*, *Anolis carolinensis*, either *Xenopus tropicalis* or *Xenopus laevis*, *Latimeria chalumnae*, either *Fugu rubripes or Danio rerio*, *Branchiostoma lanceolatum*, *and Strongylocentrotus purpuratus*. The alignments were characterized using the Protpars routine of Phylogeny Inference Package version 3.2 software (PHYLIP) [31] to make a maximum parsimony estimate of the number of substitutions that occurred along each clade of the underlying phylogeny. The sequence alignment, or updated versions thereof, is available at the Align Grantham variation Grantham deviation (Align-GVGD) website [32]. Missense substitutions observed during our mutation screening of the three MRN genes were scored using the Align-GVGD and Sorting Intolerant from Tolerant (SIFT) software programs with our curated alignments, and with polymorphism phenotyper (PolyPhen-2.1) software using its precompiled alignments [33-35]. In brief, Align-GVGD grades missense substitutions against a protein multiple sequence alignment based on a combination of Grantham Variation (GV), which measures the amount of physicochemical variation at a particular position in the alignment, and Grantham Deviation (GD), which measures the physicochemical difference between the missense residue and the range of variation observed at its position in the protein. The classifier provides seven ordered grades (C65, C55, C45, C35, C25, C15 and C0) ranging from the most likely deleterious to least likely deleterious [33]. SIFT is a sequence homology-based tool that predicts variants in the query sequence as ‘tolerated’ or ‘deleterious’ using normalized probabilities calculated from the input multiple sequence alignment [34]. Variants at a position with normalized probabilities less than 0.05 are predicted deleterious and predicted neutral with a probability greater than or equal to 0.05. PolyPhen-2 predicts variants as ‘benign’, ‘possibly damaging’, or ‘probably damaging’ based on eight sequence-based and three structure-based predictive features [35]. The alignment pipeline used in PolyPhen-2 selects homologous sequences using a clustering algorithm and then constructs and refines the alignment yielding an alignment containing both orthologs and paralogs that may or may not be full length, which yields a wider breadth of sequences but decreased depth compared with the curated alignments used with Align-GVGD and SIFT [36].

### *In silico* prediction on splicing

Sequence variants falling in the first three bp or last three bp of an exon, plus intronic variants detected in the vicinity of the splice junction sequences, with allele frequencies <1%, were scored for their potential impact on splicing using MaxEntScan (MES), which computes the maximum entropy score of a given sequence using splice site models trained on human data [37]. In work to be published elsewhere, we calibrated MES by calculating the average and standard deviation of MES scores for the wild-type splice junctions in *BRCA1*, *BRCA2*, and *ATM*, allowing us to convert raw MES scores into z-scores. Based on *BRCA1* and *BRCA2* mutation screening data used previously to calibrate Align-GVGD [33,38], we found that rare variants that fall within the acceptor or donor region and reduce the MES score for the splice signal in which they fall show an approximately 95% probability to damage splice junction function when they result in a calibrated MES score of z < −2, or approximately 40% probability when they result in a calibrated MES score of −2 < z ≤ −1 [39]; Vallee *et al*., manuscript in preparation. Thus these MES-based rules were used to identify rare sequence variants that are likely to alter MRN gene mRNA splicing.

### Analysis of rare variant threshold frequency from *ATM*, *BRCA1*, *BRCA2*, and *CHEK2* data

For *ATM*, we examined the relationship between sequence variant frequency and breast cancer risk as follows. Frequencies for all of the variants included in our 2009 *ATM* case-control mutation screening meta-analysis [19] were extracted from the EVS (for Caucasian American and African American frequencies) or UK National Genetics Reference Laboratory Manchester (NGRL Manchester) summary of 1000G data (for East Asian allele frequencies) [40]. T + SJV (as defined in [19]) plus missense substitutions and in-frame indels falling from position 1960 through the end of the protein were then grouped into the following allele frequency bins: 1% to 0.32%, 0.32% to 0.10%, 0.10% to 0.032%, <0.032%. ORs for each bin were then estimated by logistic regression, adjusting for study, ethnicity and mutation-screening method employed, using Stata version 11 software (StataCorp, College Station, TX, USA).

*CHEK2* sequence variants included in our 2011 *CHEK2* case-control mutation screening study [20] were evaluated in the same way except that (a) position in the protein was not considered, and (b) logistic regressions were adjusted for study center and race/ethnicity.

For *BRCA1* and *BRCA2*, we examined the relationship between frequency and risk as follows. First we defined ‘pseudo-cases’ and ‘pseudo-controls’ from the tested population at Myriad Genetics in the data set we used to evaluate more than 1,000 *BRCA1*/*2* sequence variants in 2007 [38]. The data set included results from approximately 68,000 full-sequence tests. Pseudo-cases for *BRCA1* were defined to be all tested individuals who were affected with breast cancer and were not found to carry a pathogenic variant in either *BRCA1* or *BRCA2*; pseudo-controls were taken to be affected individuals who were found to carry pathogenic mutations in *BRCA2* (thus explaining their personal and family history of cancer). A similar approach was taken for *BRCA2* variants, with carriers of *BRCA1* pathogenic variants serving as pseudo-controls. For each gene, variants were categorized into frequency bins as described above. Logistic regression was then used to estimate ORs for each frequency bin. Note that, because carriage of clearly pathogenic variants was used to differentiate between pseudo-cases and pseudo-controls, the *BRCA1* and *BRCA2* ORs were estimated from the distributions of unclassified variants.

### Statistical analyses of MRN case-control mutation screening data

To assess the relationship between MRN variants and breast cancer risk, analyses were performed using the chi-square test and multivariable unconditional logistic regression using Stata version 11 software (StataCorp). Differences in the case-control ratio between racial/ethnic groups and study center were accounted for by including categorical variables for each racial/ethnic group and each study center. Adjustment for racial/ethnic group should also capture confounding of genetic and social factors with interaction terms, allowing that this confounding effect may be different for the broadly labeled racial/ethnic groups in different centers. *P* values reported from analyses of the MRN case-control data are from the likelihood ratio test, adjusted for racial/ethnic group and study center, unless otherwise noted.

## Results

### Number of subjects included in the analysis

Of the 2,436 BCFR participants, three (one case from the Canadian BCFR and two controls from the Australian BCFR) were excluded because their PCR failure rate for MRN mutation-screening amplicons was greater than 20%. The distributions of the remaining 1,312 cases and 1,121 controls by age, race or ethnicity, and study center are detailed in Table 1.

**Table 1:** **Distribution of cases and controls by age, race or ethnicity, and study center**^**†**^

**Distributions**	**Cases, n (%)**	**Controls, n (%)**
Age range, yr		
≤30	108 (8.2%)	67 (6.0%)
31-35	325 (24.8%)	172 (15.3%)
36-40	436 (33.2%)	237 (21.1%)
41-45	443 (33.8%)	203 (18.1%)
46-50	0 (0.0%)	230 (20.5%)
51-55	0 (0.0%)	212 (18.9%)
Total	1,312 (100.0%)	1,121 (100.0%)
Race or ethnicity		
Caucasian	848 (64.6%)	967 (86.3%)
East Asian	208 (15.9%)	71 (6.3%)
Latina	158 (12.0%)	47 (4.2%)
Recent African Ancestry	98 (7.5%)	36 (3.2%)
Total	1,312 (100.0%)	1,121 (100.0%)
Study center		
BCFR-Australia	593 (45.2%)	522 (46.6%)
BCFR-Canada	302 (23.0%)	463 (41.3%)
BCFR-Northern California	417 (31.8%)	136 (12.1%)
Total	1,312 (100.0%)	1,121 (100.0%)

^†^The three subjects excluded because of poor mutation-screening performance are not included; percentage data are the percentages of the total number of patient or control DNA in the category indicated that met the mutation-screening quality control criterion; BCFR, Breast Cancer Family Registry.

### Relationship between frequency and odds ratio for homologous recombination repair pathway breast cancer susceptibility genes

In our case-control mutation screening studies of *ATM*, *CHEK2*, *RAD51*, and *XRCC2*, we excluded from statistical analysis sequence variants with allele frequencies above an arbitrarily selected frequency of 0.5%. With the availability of 1000G and EVS mutation screening data, it is now possible to use external data to bin rare sequence variants into allele frequency categories and then, using independent observational data, estimate odds ratio as a function of frequency. Results of such an analysis of sequence variants from confirmed breast cancer susceptibility genes, for example, *ATM*, *BRCA1*, *BRCA2*, and *CHEK2* are summarized in Table 2 and provide the basis for an empirically determined allele frequency threshold.

**Table 2:** Frequency vs odds ratio for confirmed HRR breast cancer susceptibility genes

**Gene**	**Frequency bins (%) 1.000 - 0.320 OR (95% CI)**	**0.320 - 0.100 OR (95% CI)**	**0.100 - 0.032 OR (95% CI)**	**0.032 - 0.000 OR (95% CI)**
ATM^¥^	0.00^¢^	0.57 (0.12-2.80)	1.13 (0.30-4.24)	**2.61 (1.57-4.35)**
BRCA1^§^	no variants	no variants	no variants	**2.06 (1.06-4.57)**
BRCA2^§^	no variants	0.96 (0.64-1.43)	1.60 (0.95-2.70)	**1.57 (1.15-2.15)**
CHEK2^†^	0.99 (0.20-4.91)	**3.40 (1.22-9.47)**	1.68 (0.29-9.78)	**3.31 (1.49-7.37)**

**Boldface** within the table indicates *P* <0.05. ^¥^Underlying data from [19]. Analyses were performed on data from the nine *bona fide* case-control studies. Missense substitutions were only included if they fell after position 1960 in the protein sequence. Odds ratios adjusted for study, ethnicity and mutation-screening method employed. ^¢^This grouping included just one missense substitution carried by just one control subject. ^§^Underlying data from [33]. ^†^Underlying data from [20]; odds ratios adjusted for study center and race/ethnicity.

In the 0.32% to 0.10% allele frequency bin, there was significant evidence of pathogenic variants in *CHEK2*; indeed, the known pathogenic *CHEK2* variants c.1100delC (Chr22:29091856delG) and p.Ile157Thr (rs17879961) both have allele frequencies between 0.32% and 0.10% in the EVS ‘Caucasian American’ sample. But there was no evidence for pathogenic variants in the other three genes in this allele frequency range.

In the 0.10 to 0.032% bin, *BRCA2* had an OR of 1.60 (*P* = 0.078). Although just shy of significant, an elevated *BRCA2* OR in this EVS frequency bin is supported by the presence of known pathogenic *BRCA2* variants with multiple reports in the Breast Information Core (BIC) database such as c.2806_2809delAAAC (rs80359351) and c.3847_3848delGT (rs80359405) [41]. While the OR for *ATM* in this frequency bin was very near 1.0, our data set did contain one known pathogenic in-frame deletion in the gene, p.RIS2547_2549del (c.7638_7646del9), which was observed in a control.

Below an allele frequency of 0.032%, all four genes had significant evidence of pathogenic variants. We note that, in genotype-phenotype terms, the MRN genes more closely resemble *BRCA1*, *BRCA2*, and *ATM* than *CHEK2* in that inheritance of biallelic mutations in the MRN genes is either embryonic lethal or causes a developmental phenotype that severely reduces reproductive fitness; we know of no such evidence for biallelic *CHEK2* mutation carriers [42-44]. Therefore, noting that there was no evidence for pathogenic variants in *ATM*, *BRCA1*, or *BRCA2* with allele frequencies >0.1% in continental level populations, we set our threshold for evaluating rare MRN variants at an allele frequency ≤0.1% in Caucasian Americans, African Americans, and East Asians, based on EVS data for the former two groups and 1000G data, as summarized by the NGRL Manchester, for East Asian allele frequencies [40].

### Analyses of rare silent substitutions and rare, analytically innocuous splice junction variants

Full open reading frame mutation screening of the MRN genes revealed 20 rare silent substitutions and 21 splice junction variants that look innocuous by the MaxEntScan based sequence analysis criteria described in the Methods (Table S1 in Additional file 1). Accounting for subjects who carried two rare variants, 10 cases and 10 controls carried a rare silent substitution and no other potentially more severe rare variant, resulting in an OR of 0.95 (*P* = 0.91). Similarly, 14 cases and 13 controls carried an innocuous rare splice junction variant and no other potentially more severe rare variant, resulting in an OR of 0.62 (*P* = 0.25) (Table 3).

**Table 3:** Analyses of largely innocuous groups of rare variants

**Class**	**Cases, n**	**Controls, n**	**Crude OR (95% CI)**	**Adjusted OR**^**† **^**(95% CI)**
Noncarriers	1,240	1,060		
Silent	10	10	0.85 (0.35-2.06)	0.95 (0.38-2.39)
Splice	14	13	0.92 (0.43-1.96)	0.62 (0.28-1.39)
Any rMS or in-frame indel^¥^	48	38	1.08 (0.70-1.67)	0.96 (0.61-1.50)

^†^In these binary logistic regressions, the regression coefficient = ln(OR). ^¥^Includes the carrier of the *RAD50* final exon frameshift c.3852del4, which we treat as an in-frame deletion because it should not cause nonsense-mediated decay. One subject (a case) carried both a rare innocuous splice donor variant and a rare silent substitution. For analyses summarized in this table, this subject was categorized as a carrier of an innocuous splice junction variant. Four subjects (one case and three controls) carried both a rare missense substitution and a rare silent substitution. For analyses summarized in this table, these subjects were categorized as carriers of a rare missense substitution. One subject (a case) carried both a rare missense substitution and a rare innocuous splice acceptor variant. For analyses summarized in this table, this subject was categorized as a carrier of a rare missense substitution. Two subjects (one case and one control) carried two rare missense substitutions. In this table, these subjects were categorized as carriers of a rare missense substitution. OR, odds ratio; CI, confidence interval.

### Analysis of protein-truncating variants

Full open reading frame mutation screening of the MRN genes revealed three nonsense substitutions, five frameshift variants, one severely damaging splice donor variant, one severely damaging splice acceptor variant, and two moderately damaging splice donor variants (Table 4 and Table S1 in Additional file 1). Of these 12 variants, only one, *RAD50* c.2938del5, was carried by more than one subject (two controls). In addition, one of these 12 variants, *RAD50* c.3852del4, falls in a final coding exon where it would not trigger nonsense-mediated decay. Analytically, we considered this as being analogous to an in-frame deletion and included the variant in our analyses of rMS and in-frame deletions rather than our analysis of T + SJV. With nine T + SJV observations in cases against three in controls, the OR for T + SJV was 2.61, *P* = 0.14 (Table 5).

**Table 4:** Severity scores applied to selected classes of potentially pathogenic rare variants

**Class**	**Binary**	**Graded**	**#Variants**	**Cases, n**	**Controls, n**
	**severity**	**severity**			
Truncating and spliceogenic variants					
Frameshift, excluding the last exon	1.0	6.0	4	2	3
Nonsense, excluding the last exon	1.0	6.0	3	3	0
Severe acceptor damage	1.0	5.7	1	1	0
Moderate acceptor damage	1.0	2.4	0	0	0
Severe donor damage	1.0	5.7	1	1	0
Moderate donor damage	1.0	2.4	2	2	0
Missense					
Key domain rMS, graded C0	0.0	0.0	4	4	1
Key domain rMS, graded C15	1.0	1.0	1	1	0
Key domain rMS, graded C25	1.0	2.0	6	6	1
Key domain rMS, graded C35	1.0	3.0	1	0	1
Key domain rMS, graded C45	1.0	4.0	3	3	0
Key domain rMS, graded C55	1.0	5.0	0	0	0
Key domain rMS, graded C65	1.0	6.0	8	10	1
Key domain in-frame deletion^†^	1.0	6.0	1	0	1

^†^Includes the *RAD50* final exon truncating variant c.3852del4, which falls within the C-terminal ATPase domain (a key domain), which we consider analytically equivalent to a key domain rMS graded C65. rMS, rare missense substitution.

**Table 5:** Analyses of potentially pathogenic groups of rare variants

**Class**	**Cases, n**	**Controls, n**	**Crude OR**^**† **^**(95% CI)**	**Adjusted OR**^**† **^**(95% CI)**
Noncarriers	1,283	1,114		
T + SJV^¥^				
MRE11A	1	0		
RAD50^$^	4	3		
NBN	4	0		
Total	9	3	2.60 (0.70-9.65)	2.61 (0.67-10.1)
Any key functional domain rMS or in-frame indel^*^				
MRE11A^¢^	10	1		
RAD50	10	2		
NBN	4	2		
Total	24	5	**4.18 (1.59-11.0)**	**3.17 (1.17-8.59)**
Key functional domain rMS (severity > C0) or in-frame indel^*^				
MRE11A^¢^	7	1		
RAD50	10	2		
NBN	3	1		
Total	20	4	**4.34 (1.48-12.7)**	**3.07 (1.01-9.31)**
T + SJV^¥^ plus key functional domain rMS (severity > C0) or in-frame indel^*^				
MRE11A^¢^	8	1		
RAD50^$^	14	5		
NBN	7	1		
Total	29	7	**3.60 (1.57-8.24)**	**2.88 (1.22-6.78)**

**Figure 1:**
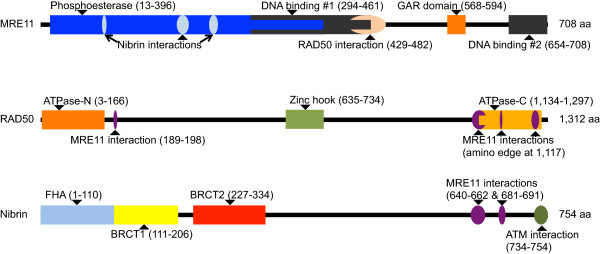
**MRE11, RAD50, and nibrin key functional domains.** The protein domain diagrams are updated from Williams *et al*. [4] and include information from the InterPro protein sequence analysis and classification database, the Uniprot Protein Knowledgebase, and the NCBI Conserved Domains Database [47-50] plus structural studies of the MRN proteins [51-63].

**Boldface** within the table indicates *P* <0.05. ^†^In these binary logistic regressions, the regression coefficient = ln(OR). ^¥^Truncating and splice junction variants; excludes final exon nonsense and frameshift variants. ^$^One subject (a case) carried both the *RAD50* splice acceptor variant RAD50_c.552-1G > A and the *RAD50* silent substitution RAD50 c.3153G > A (p.L1051L). ^*^The key functional domains are defined in Figure 1. The set of variants includes rare missense substitutions with A-GVGD scores > C0, and final exon nonsense and frameshift variants if they also fall in a key functional domain. ^¢^One subject (a case) carried both the *MRE11A* key domain rare missense substitution MRE11A p.D235G and the *NBN* non key domain rare missense substitution NBN p.V210F. OR, odds ratio; CI, confidence interval.

### Analyses of rare missense substitutions and in-frame indels

We observed 58 distinct rMS, one in-frame deletion, and one final exon frameshift (*RAD50* c.3852del4) that we treated as an in-frame deletion (Table S1 in Additional file 1). Taking into account that two subjects (one case and one control) carried two rMS, these added up to 48 cases and 38 controls that carried one or more rMS or in-frame indel. The OR for this class of variants was 0.96 (*P* = 0.85) (Table 3).

In our analyses of *BRCA1* and *BRCA2,* we found that evidence for pathogenic, non-spliceogenic, missense substitutions in those genes was limited to substitutions that fall in the protein’s key functional domains and have severity scores of greater than C0 when evaluated by Align-GVGD [33,38]; that observation is also supported by an extensive functional assay analysis of *BRCA1* missense substitutions [45]. In our case-control mutation screening analysis of *ATM*, we also found that evidence for rMS that predispose to breast cancer was limited to rMS with Align-GVGD severity scores of greater than C0 that fall in the key functional domains of the protein that are central to its enzymatic activity [19]. Accordingly, we carried out an analysis of MRN gene rMS limited to these key functional domains, largely as described in a recent review of MRN protein structure and function [4] with the exception that we excluded from the analysis variants falling in the second DNA binding domain of MRE11A because this domain is required for DSB formation during meiosis but not for repair of DSBs [46].

Limited to the key functional domains as annotated on Figure 1 (which is derived from Williams *et al*. [4]), and excluding the second DNA binding domain of MRE11A, we observed 23 rMS and one final exon truncating variant that we evaluate as an in-frame deletion (RAD50 c.3852del4). These variants were carried by 24 cases and five controls, resulting in an OR of 3.17 (*P* = 0.012) (Table 5 and Table S1 in Additional file 1).

To refine analyses of the key domain rMS, we prepared and hand-curated protein multiple sequence alignments of the MRN proteins. Each alignment contains 16 sequences from MRN orthologs and, from the perspective of the position of *Homo sapiens* in the vertebrate phylogeny, each samples the same key nodes of mammalian and vertebrate evolution. The concatenated alignment slightly exceeds an average of 3.0 substitutions per position, reaching our criterion of sufficient sequence diversity for evaluating missense substitutions [36]. Using these alignments, the key domain rMS were scored with Align-GVGD and SIFT; PolyPhen-2 scores for these variants were also extracted from PolyPhen’s pre-computed exome-wide data set [32,64,65]. The key domain final exon truncating variant *RAD50* c.3852del4 was assigned Align-GVGD, SIFT, and PolyPhen-2 scores corresponding to the most severe missense substitution created by the frameshifted coding; these were C65, 0.00, and 0.996, respectively (Align-GVGD and PolyPhen-2 scales have the opposite polarity of the SIFT scale; all three of these scores are indicative of an extremely damaging variant). Accordingly, for all further analyses described here, *RAD50* c.3852del4 is included in the most severe grade of key domain rMS unless otherwise noted.

A conservative view of protein multiple sequence alignment-based evaluation of human missense substitutions is that each missense substitution that falls within or very close to the range of variation observed at its position in an appropriately informative alignment should be neutral or nearly so. With Align-GVGD, this would correspond to missense substitutions that score C0. Using the complete alignments of the MRN proteins, 11 of the key domain rMS scored C0; these were carried by 11 cases and just one control, potentially indicative of overly stringent alignment depth. Reducing the stringency of the Align-GVGD scoring by restricting the alignments to the mammals-only subset (comprising sequences from nine species), four of the key domain rMS scored C0, and these were carried by four cases and one control. Excluding these four substitutions as likely innocuous, the 20 remaining key domain rMS were carried by 20 cases and four controls (Table 5 and Table S1 in Additional file 1). The OR for the non-C0 key domain rMS and in-frame deletions was 3.07 (*P* = 0.029).

Exploiting the intrinsic ordering of the seven Align-GVGD grades from C0 to C65, we performed a logistic regression test for log-linear OR trends across noncarriers and carriers of the seven grades of rMS. In this test, the C0 substitutions are again considered very likely neutral and assigned a severity grade of 0, which was the same as the grade assigned to noncarriers. The more severe grades were assigned sequentially higher severities, with C65 substitutions assigned a severity grade of 6 (Tables 4 and 6). The test yielded a lognormal OR increase of 0.21/grade (*P* = 0.077), which corresponds to a modeled OR of 3.60 at grade 6 (Table 6).

**Table 6:** Graded tests

**Class**	**Adjusted?**	ln(OR) regression coefficient^* ^**(95% CI)**	**Modeled OR at most severe grade**^**† **^**(95% CI)**	**Observed OR at most severe grade**^**¥ **^**(95% CI)**
MRN Key Domain missense, graded^$^	crude	**0.30 (0.055-0.55)**	**6.16 (1.39-27.3)**^¢^	4.31 (0.94-19.7)^¢^
	adjusted	0.21 (−0.052-0.48)	3.60 (0.73-17.7)^¢^	1.80 (0.38-8.44)^¢^
MRN T + SJV, graded	crude	0.14 (−0.088-0.37)	2.34 (0.59-9.34)^¢^	2.03 (0.52-7.86)^¢^
	adjusted	0.15 (−0.091-0.38)	2.41 (0.58-10.0)^¢^	2.15 (0.53-8.78)^¢^
MRN T + SJV + KeyD missense, graded^$^	crude	**0.23 (0.059-0.39)**	**3.90 (1.42-10.7)**^¢^	**2.93 (1.08-7.97)**^¢^
	adjusted	**0.18 (0.0011-0.35)**	**2.89 (1.01-8.30)**^¢^	1.97 (0.69-5.62)^¢^
MRN T + SJV + KeyD missense, graded^$^ (C65 rMS and in-frame indel removed)	crude	**0.31 (0.0094-0.62)**	**3.50 (1.04-11.8)**^**∞**^	2.88 (0.79-10.5)^**∞**^
	adjusted	**0.33 (0.028-0.64)**	**3.79 (1.12-12.9)**^**∞**^	3.43 (0.91-12.9)^**∞**^

**Boldface** within the table indicates *P* <0.05. ^*^From a standard logistic regression of form ln(OR) = a + b(x) in which a = 0, b is the logistic regression OR trend coefficient, and x is, in this case, sequence variant grade. Note that the regression coefficient is significant if its 95% CI excludes 0.00. ^†^This is the OR calculated for the most severe grade included in the logistic regression, using the ln(OR) regression coefficient. ^¥^This is the OR point estimate for carriers of the most severe grade included in the corresponding graded regression model. ^$^The key functional domains are defined in Figure 1. The set of variants includes final exon nonsense and frameshift variants if they also fall in a key functional domain. ^¢^This is a six-grade regression with the most severe grade consisting of key domain C65 rMS and in-frame indels, truncating variants, and severely damaging splice junction variants. ^∞^With C65 rMS and the in-frame indel removed, and with no C55 rMS in the data set (Table 4), this is a four-grade regression with the most severe grade consisting of key domain C45 rMS, truncating variants, and severely damaging splice junction variants. OR, odds ratio; CI, confidence interval.

### Truncating variants and rare missense substitutions assessed in a single model

Using a simple binary classification to combine T + SJV plus key functional domain rMS of grade > C0 into a single model, we observed 29 carriers among the cases against seven among the controls. This binary classification resulted in an OR of 2.88, *P* = 0.0090 (Table 5).

To add the T + SJV into the regression test for OR trend across the graded rMS, we assigned protein-truncating variants a severity grade equal to that of the highest grade rMS included in that regression, in this case 6.0. Potentially damaging splice junction variants were assigned this severity grade x their probability to damage a splice junction, that is, severely damaging splice junction variants were assigned a severity grade of 0.95 × 6 = 5.7, and moderately damaging splice junction variants a severity grade of 0.40 × 6 = 2.4. This test yielded a lognormal OR increase of 0.18/grade (*P* = 0.033), which corresponds to a modeled OR of 2.89 at the most severe grade (Table 6).

In addition to the lognormal OR coefficient and modeled OR at the most severe sequence variant grade, Table 6 also reports the OR point estimate at the most severe sequence variant grade. One unusual result was that, for the analysis or rMS, the logistic regression adjustment for race/ethnicity and study center decreased the modeled OR for key domain C65 rMS by 42% and decreased the OR point estimated for this class of rMS by 58% compared to the crude result. These ORs also decreased in the combined test of rMS plus T + SJV, but not as dramatically.

Looking at the underlying data, 11 of the 12 observations of key domain C65 rMS - including both controls that carried such a variant - were carried by either an East Asian or a Latina. Because the case:control ratio in these groups was 3:1 (Table 1), the adjustment for race/ethnicity applies a relatively high weight to the genotypes of the East Asian and Latina controls, explaining the marked decrease in the OR point estimates upon adjustment. As clustering of C65 rMS in the East Asian and Latina subjects was an unexpected heterogeneity in our data set, we checked for heterogeneity across racial/ethnic groups by class of likely pathogenic sequence variant (Table 7). While there was no evidence for heterogeneity in the distributions of T + SJV or rMS in general, subset analysis found racial/ethnic heterogeneity across the key domain rMS (*P* = 0.003) and sub-subset analysis found that this heterogeneity was localized exclusively to the key domain C65 variants (*P* = 0.96 for rMS of grade C0 to C55, but *P* <1x10^−5^ for C65 rMS). To examine the impact of racial/ethnic heterogeneity in the distribution of key domain C65 rMS on the evidence in favor of the MRN genes as intermediate-risk breast cancer susceptibility genes, we assessed the key domain C65 missense substitutions alone and re-ran the binary analysis of T + SJV and key domain rMS with the C65 variants excluded (Table 8). The crude OR for key domain C65 missense substitutions alone was 4.3, but dropped to 1.78 upon adjustment for race/ethnicity and study center. Nonetheless, excluding these C65 rMS from the binary analysis of T + SJV and key domain rMS resulted in an OR of 3.39 (Table 5), and the *P* value (*P* = 0.010) was virtually identical to that observed when the C65 rMS were included (*P* = 0.0090). Similarly, setting aside the issue of racial/ethnic heterogeneity by limiting the binary analysis of T + SJVs and key domain rMS to Caucasians of European ancestry resulted in an OR of 4.08 (*P* = 0.0068) (Table 8). Finally, exclusion of the key domain C65 rMS from the graded test of rMS and T + SJV resulted in a lognormal OR increase of 0.33/grade (*P* = 0.021) and virtually no change in the OR coefficient upon adjustment (Table 6). Thus the racial/ethnic heterogeneity in the distribution of these rMS does not impinge on the overall result of this study. Of note, among the key domain C65 rMS, the *RAD50* zinc hook domain missense substitution p.Arg725Trp was observed in three Latina cases; the EVS reports that the variant was observed in 1 of 2,200 African Americans and the variant was not present in 1000G data.

**Table 7:** Tests of heterogeneity

**Class**	**Crude (Fisher’s exact)**	**Adjusted**
Heterogeneity across racial/ethnic groups by sequence variant class		
Racial/ethnic group: T + SJV	0.11	NA^¥^
Racial/ethnic group: any rMS	0.35	NA^¥^
subset: non-key domain rMS^*^:	0.40	NA^¥^
subset: key domain rMS^*^:	0.003	NA^¥^
sub-subset: key domain rMS^*^, <C65	0.96	NA^¥^
sub-subset: key domain rMS^*^, C65	<1x10^−5^	NA^¥^
Heterogeneity across genes, by likely pathogenic sequence variant class		
MRN: T + SJV	0.43	NC^†^
MRN: key domain rMS^*^	1.00	0.68
MRN: rare T + SJV and key domain rMS^*^	0.53	0.30

^¥^We have adjusted most *P* values for racial/ethnic group and study center. But a test for heterogeneity across racial ethnic groups cannot be adjusted for racial/ethnic group; moreover, as most of the subjects of East Asian, Latina, and Recent African Ancestry were obtained from one study center (the Cancer Prevention Institute of California), we cannot adjust these tests on that variable, either. ^†^Adjusted *P* value could not be calculated because some cells contained 0. ^*^The key functional domains are defined in Figure 1. The set of variants includes rare missense substitutions with A-GVGD scores > C0, and final exon nonsense and frameshift variants if they also fall in a key functional domain. T + SJV, truncating and splice junction variants; rMS, rare missense substitution; MRN, MRE11-RAD50-NBN complex.

**Table 8:** Impact of race/ethnicity heterogeneity in distribution of rare key domain C65 missense substitutions

**Class**	**Cases, n**	**Controls, n**	**Crude OR**^**† **^**(95% CI)**	**Adjusted OR**^**† **^**(95% CI)**
Noncarriers	1,283	1,114		
Key functional domain^*^ C65 rMS or in-frame indel^§^				
*MRE11A*	4	0		
*RAD50*	4	1		
*NBN*	2	1		
Total	10	2	4.35 (0.95-19.9)	1.78 (0.38-8.35)
Key functional domain^*^ rMS (C0 < severity < C65)^$^				
*MRE11A*	3	1		
*RAD50*	6	1		
*NBN*	1	0		
Total	10	2	4.34 (0.95-19.9)	4.55 (0.97-21.3)
T + SJV^¥^ plus key functional domain^*^ rMS (C0 < severity < C65)^$^				
*MRE11A*	4	1		
*RAD50*	10	4		
*NBN*	5	0		
Total	19	5	**3.30 (1.23-8.87)**	**3.39 (1.23-9.33)**
T + SJV^¥^ plus key functional domain^*^ rMS (severity > C0), analysis limited to CEU^¶^ subjects.				
*MRE11A*	3	0		
*RAD50*	7	4		
*NBN*	4	0		
Total	14	4	**4.04 (1.33-12.3)**	**4.08 (1.32-12.5)**

**Boldface** within the table indicates *P* <0.05. ^†^In these binary logistic regressions, the regression coefficient = ln(OR). ^*^The key functional domains are defined in Figure 1. ^§^Eight distinct key functional domain C65 missense substitutions and the *RAD50* final exon frameshift c.3852del4 fall in this group (Table 4). ^$^Eleven distinct key functional domain missense substitutions fall in this group (Table 4). ^¥^Truncating and splice junction variants; excludes final exon nonsense and frameshift variants. ^¶^Limiting to CEU (Caucasians of European ancestry) subjects reduces the number of case and control noncarriers to 834 and 963, respectively. rMS, rare missense substitution; T + SJV, truncating and splice junction variants.

## Discussion

In the present work, we evaluated the contribution of rare variants in the genes *MRE11A*, *RAD50*, and *NBN* to breast cancer risk. As the proteins encoded by these genes form an evolutionarily conserved complex that could be functionally impaired by a dysfunctional variant in any one of the genes, we evaluated them as if they constitute a single candidate susceptibility gene. Combining T + SJV, and key functional domain rMS, we found that this set of rare MRN genes variants contributes to breast cancer susceptibility (OR = 2.88, *P* = 0.0090). A *post hoc* test for heterogeneity did not reveal evidence for between-gene differences in the case-control distributions of likely pathogenic variants: Fisher’s exact test *P* values of between-gene heterogeneity for T + SJV, key domain rMS and the combination of the two classes of rare variants were 0.43, 1.00, and 0.53, respectively (Table 7). Similarly, looking at the genes individually, neither truncating variants, nor key domain missense substitutions, nor a combination of the two reached statistical significance from single gene data (Table 9). Thus evidence from this study in favor of the MRN genes as intermediate-risk breast cancer susceptibility genes emerges from the ensemble analysis of the three genes.

**Table 9:** **Individual contributions of *MRE11A*, *RAD50*, and *NBN *** **to the ensemble model**

**Class**	**Cases, n**	**Controls, n**	**Crude OR**^**† **^**(95% CI)**	**Adjusted OR**^**† **^**(95% CI)**
Noncarriers	1,283	1,114		
MRE11A				
T + SJV^¥^	1	0	^∞^[*P* = 1.00]^¥^	
rMS or IFD^*¢^	7	1	6.08 (0.75-49.5)	3.62 (0.42-31.5)
Combined	8	1	6.95 (0.87-55.6)	5.02 (0.59-42.8)
RAD50				
T + SJV^¥$^	4	3	1.16 (0.26-5.18)	1.09 (0.23-5.24)
rMS or IFD^*^	10	2	4.34 (0.95-19.9)	3.21 (0.68-15.2)
Combined	14	5	2.43 (0.87-6.77)	1.98 (0.68-5.71)
NBN				
T + SJV^¥^	4	0	^∞^[*P* = 0.13]^¥^	
rMS or IFD^*^	3	1	2.60 (0.27-25.1)	2.12 (0.20-22.6)
Combined	7	1	6.08 (0.75-49.5)	5.28 (0.62-45.2)
MRE11A, RAD50, and NBN ensemble model^*^				
T + SJV^¥$^	9	3	2.60 (0.70-9.65)	2.61 (0.67-10.1)
rMS or IFD^*¢^	20	4	**4.34 (1.48-12.7)**	**3.07 (1.01-9.31)**
Combined	29	7	**3.60 (1.57-8.24)**	**2.88 (1.22-6.78)**

**Boldface** within the table indicates *P* <0.05. ^†^In these binary logistic regressions, the regression coefficient = ln(OR). ^¥^Truncating and splice junction variants; excludes final exon nonsense and frameshift variants. ^¥^Because there were no observations of this class of variants in the controls, the crude odds ratio is infinity (∞) and the *P* value was calculated by Fisher exact test rather than by logistic regression; this also precludes calculation of adjusted odds ratios. As 95% confidence intervals could not be estimated, we report the *P* value. ^*^Key functional domain rare missense substitutions with A-GVGD scores > C0, in-frame indels, and final exon nonsense and frameshift variants if they also fall in a key functional domain. The key functional domains are defined in Figure 1. ^¢^One subject (a case) carried both the *MRE11A* key domain rare missense substitution MRE11A p.D235G and the *NBN* non key domain rare missense substitution NBN p.V210F. ^$^One subject (a case) carried both the *RAD50* splice acceptor variant *RAD50* c.552-1G > A and the *RAD50* silent substitution RAD50 c.3153G > A (p.L1051L). T + SJV, truncating and splice junction variants; rMS, rare missense substitution; IFD, in-frame deletion.

Although MRN gene T + SJV were not by themselves a significant breast cancer risk factor, we note that our OR point estimate of 2.61 is both very close to the meta-analysis point estimate of 2.63 that Zhang *et al*. obtained for *NBN* c.657del5 [13], and close to the point estimate of 2.32 that we obtained in our meta-analysis of *ATM* T + SJV [19]. Thus, while we cannot exclude that our nonsignificant finding is actually indicative of little or no risk of breast cancer conferred by MRN gene protein-truncating variants, our data are more strongly in accord with the hypothesis that they confer an intermediate risk of magnitude similar to the risk conferred by truncating variants in *ATM*.

Overall, there was no association between rMS and risk of breast cancer. Nevertheless, tightening the focus to key functional domain rMS resulted in a significant association with an OR of approximately 3.0. In this sense, the MRN genes behave as the homologous recombination repair genes *BRCA1*, *BRCA2*, and *ATM* - genes in which rare missense substitutions that are pathogenic because of missense dysfunction *per se* are largely confined to key functional domains.

Combining MRN T + SJV and key functional domain rMS, we observed an OR of 2.88 with a *P* value of 0.0090. That *P* value meets the threshold of *P* <0.01 that Hollestelle *et al*. suggested for establishing intermediate-risk susceptibility genes that were already strong candidates based on their biochemical function [3]. Thus with a mutation screening and data analysis approach that considered MRE11A, RAD50, and Nibrin as a unique functional entity and focused the analysis of rMS to those that fall in the key functional domains of the MRN complex, we overcame the limitation of previous suggestive studies that were based on a small number of founder mutations [13,15,18], and confirmed the hypothesis that *MRE11A*, *RAD50*, and *NBN* are intermediate-risk susceptibility genes in a general sense. Moreover, because we did not observe any of the four sequence variants most responsible for the MRN genes’ candidate gene status (*MRE11A* p.Arg202Gly, *MRE11A* p.Arg633Stop, *RAD50* c.687delT, and *NBN* c.657del5) [13,14,18], this confirmation is independent of the hypothesis-generating data.

Five of the sequence variants observed in the MRN case-control mutation screening bear further discussion.

*NBN* p.Arg215Trp was of interest because association studies have found evidence that it confers modest risk of several cancers (for review, see [66]), and there is biochemical evidence, albeit somewhat conflicting, of altered function of this nibrin allele [67,68]. We observed six cases and six controls with the p.Arg215Trp missense substitution, resulting in an OR of 0.96 (95% CI 0.30 to 3.06, *P* = 0.95). While these confidence intervals are too wide to exclude the possibility that *NBN* p.Arg215Trp is actually a modest-risk variant, we also point out that position Arg215 is quite variable in our protein multiple sequence alignment and that, according to EVS data, the variant has a frequency of 0.37% in Caucasian Americans - well above the frequency threshold we found for severely dysfunctional variants in homologous recombination repair genes in which biallelic mutations cause embryonic lethality or severe childhood disease.

Second, we observed one carrier, a Caucasian control ascertained at the age of 55, of the *MRE11A* in-frame deletion c.2109del9. The variant falls in the last exon of the gene, near the carboxy terminus of the second DNA binding domain (which is also the carboxy terminus of the protein). Because this domain is required for double-strand break formation during meiosis but not for repair of double-strand breaks [46], the domain was not included in the list of ‘key functional domains’ and the indel was not included in statistical analyses of key functional domain variants.

Third, we observed one carrier, an East Asian case diagnosed at the age of 35, of *MRE11A* p.Thr481Ile. This residue is a threonine in all but one species in our alignment, but is a methionine in the cephalochordate, *Branchiostoma lanceolatum*. The substitution falls within the protein’s RAD50 interaction domain. While very few of the rare variants that we observed have been reported in human ataxia-telangiectasia-like disease or Nijmegen breakage syndrome patients, another substitution at this residue, p.Thr481Lys, was observed in an Italian ataxia-telangiectasia-like disease sib-pair [69].

Fourth, we observed one carrier, an East Asian control ascertained at age 50, of the *RAD50* frameshift c.3852del4. Because the frameshift falls in the last exon of the gene where it would not be expected to cause nonsense-mediated decay of the mRNA, we evaluated it as an in-frame deletion rather than as a frameshift. As such, it scrambles well-conserved sequence near the carboxy terminus of the protein’s carboxy-end ATPase domain and final MRE11A binding domain including positions such as Arg1288 and Lys1291 that are invariant in our protein multiple sequence alignment. The sequence scrambling creates nonconservative substitutions at invariant key functional domain positions, resulting in the highest possible sequence variant severity score.

Fifth, we observed one carrier, also an East Asian control ascertained at age 50, of the *NBN* missense substitution p.Ile35Thr. This position falls in the protein’s functionally important forkhead-associated (FHA) domain and is either isoleucine or leucine in all of the species included in our NBN protein multiple sequence alignment.

The last three variants described above illustrate two of the analytic problems encountered in this study. All three were evaluated as key domain C65 rMS and all three were observed in East Asian subjects. Combined with eight additional observations of key domain C65 rMS in either East Asian or Latina subjects against just one in a Caucasian of European ancestry, there was an unexpected excess of these variants in the non-Caucasian subjects mutation screened in this study. Second, the two variants observed in the controls affected positions with little or no cross-species physicochemical variability; consequently, they would be graded as severe C65 variants with either a mammals-only protein multiple sequence alignment or with our complete alignment through the deuterostomate *Strongylocentrotus purpuratus*. In contrast, the *MRE11A* rMS that described from a breast cancer case (p.Thr481Ile), as well as the rMS observed at the same position in a pair of ataxia-telangiectasia-like disease cases (p.Thr481Lys), score as severe C65 substitutions when evaluated with the mammals-only alignment but as likely innocuous C0 substitutions when evaluated with the evolutionarily deep alignment. Since the observation of a nonconservative rMS at *MRE11A* position Thr481 in a pair of ataxia-telangiectasia-like disease cases increases the odds that substitutions at this position are in fact pathogenic, it appears that using the evolutionarily deeper alignments is, for the MRN genes, counterproductive. On the other hand, the empirically determined allele frequency thresholds derived by combining older *ATM*, *BRCA1*, *BRCA2*, and *CHEK2* case-control mutation screening data with EVS and 1000G data - found to be 0.1% for the three genes (*ATM*, *BRCA1*, and *BRCA2*) where inheritance of biallelic mutations is either embryonic lethal or causes a developmental phenotype that severely reduces reproductive fitness, and 0.32% for *CHEK2* - provides a new tool to help with evaluation of the many rare variants observed in a case-control mutation screening study of candidate cancer susceptibility genes.

For *BRCA1* and *BRCA2*, it is well established that a strong majority of pathogenic variants are, ultimately, protein-truncating variants. In contrast, case-control mutation screening of *CHEK2* revealed an approximately equal contribution from T + SJVs and rMSs to the fraction of breast cancer attributable to rare variants in that gene, and a case-control mutation screening meta-analysis of *ATM* revealed that rMS in that gene may actually be responsible for a larger fraction of the breast cancer attributable to rare variants than are the T + SJVs [19,20]. In the mutation screening data reported here, rare key functional domain missense substitutions in the MRN genes were more frequent (24 vs. 12 observations) than truncating variants and conferred a slightly higher OR (3.07 vs. 2.61) with a lower *P* value (0.029 vs. 0.14). These data are more congruent with the *ATM*/*CHEK2* pattern than the *BRCA1/2* pattern. Since there is not yet any efficient approach to clinically actionable classification of missense substitutions in these genes, these data point toward a clinical problem. When the MRN genes are mutation screened as part of a clinical panel-based cancer susceptibility gene sequencing test, a large fraction, if not the majority, of the genetic risk attributable to them will reside in rare missense substitutions that will initially be reported to clinical geneticists as unclassified variants.

The analytic strategy of treating the three genes as a single concatenated gene had one notable drawback: we are not able to ask whether variants in each of the three genes are best evaluated under the same analysis model. Thus an enormous amount of work, likely involving larger scale mutation screening efforts to gain more analytic precision, tests of segregation to examine penetrance and tumor spectrum, and perhaps development of functional assays to aid evaluation of rare missense substitutions, remains to be performed on with *MRE11A*, *RAD50*, and *NBN*.

## Conclusions

Results reported here establish that *MRE11A*, *RAD50*, and *NBN* are intermediate-risk breast cancer susceptibility genes and help to justify their inclusion on panel-based cancer susceptibility gene tests. Protein-truncating variants and rare missense substitutions falling in the key functional domains of these proteins appear to confer two- to three-fold increased risk of breast cancer. Like *ATM* and *CHEK2*, the spectrum of pathogenic variants in the MRN genes includes a relatively high proportion of missense substitutions. However, the data neither establish whether variants in each of the three genes are best evaluated under the same analysis model nor achieve clinically actionable classification of individual variants observed in this study. Given the relatively low frequency of likely pathogenic variants in the MRN genes, development of clinically applicable rare missense substitution classification models for these genes will require data from very large observational studies supplemented, in all likelihood, by carefully calibrated functional assays.

## Abbreviations

1000G: 1000 genomes project; Align-GVGD: Align Grantham variation Grantham deviation; ATM: ataxia telangiectasia mutated; BCFR: Breast Cancer Family Registry; BIC: Breast Cancer Information Core; bp: base pair; BRCA1: Breast Cancer 1 gene; BRCA1/2: BRCA1 and/or BRCA2; BRCA2: Breast Cancer 2 gene; CHEK2: checkpoint kinase 2; CI: confidence interval; CIHR: Canadian Institutes for Health Research; DSB: double-strand breaks; EVS: exome variant server; HRM: high-resolution melting curve analysis; IARC: International Agency for Research on Cancer; IRB: Institutional Review Board; MES: MaxEntScan; MIM: Mendelian Inheritance in Man; *MRE11A*: meiotic recombination 11; MRN complex: MRE11-RAD50-NBN complex; *NBN*: nibrin; NCI: United States National Cancer Institute; NGRL Manchester: UK National Reference Laboratory Manchester; NIH: United States National Institutes of Health; OR: odds ratio; PCR: polymerase chain reaction; PHYLIP: Phylogeny Inference Package; PolyPhen: polymorphism phenotyper; rMS: rare missense substitution; SIFT: Sorting Intolerant from Tolerant; SNP: single-nucleotide polymorphism; T- or M-Coffee: Tree-based consistency objective function for alignment evaluation; T + SJV: truncating and splice junction variants; WGA DNA: whole-genome amplified deoxyribonucleic acid; XRCC2: X-ray cross complemention group 2.

## Competing interests

The authors declare that they have no competing interests.

## Authors’ contributions

FD led the mutation screening of *RAD50*, contributed to data analysis, and helped to draft the manuscript. MP led the mutation screening of *NBN*, contributed to data analysis, and helped to draft the manuscript. JO led the mutation screening of *MRE11A*, contributed to data analysis, and helped to draft the manuscript. FLCK contributed to study design, led the laboratory team, and helped to draft the manuscript. CV was responsible for data management throughput for the project, helped to refine the laboratory platform, and helped to draft the manuscript. ELY built and curated the MRN protein multiple sequence alignments, helped with scoring missense substitutions, and helped to draft the manuscript. NR contributed to the mutation screening and data analysis, helped to refine the laboratory platform, and helped to draft the manuscript. NF contributed to the mutation screening and data analysis, helped to refine the laboratory platform, and helped to draft the manuscript. GD contributed to the mutation screening and data analysis, helped to refine the laboratory platform, and helped to draft the manuscript. MPV built the algorithm for evaluating splice junction variants and helped to draft the manuscript. KT contributed to evaluation of splice junction variants and helped to draft the manuscript. TCR adapted the splice junction analysis algorithm to run on sequence variants written in genome coordinates and helped to draft the manuscript. GJW defined the coordinates of the MRN protein key functional domains and helped to draft the manuscript. JLH was the lead investigator for subjects gathered through the Australian site of the BCFR and helped to draft the manuscript. MCS contributed to study design, contributed to the management of samples obtained through the Australian site of the BCFR, and helped to draft the manuscript. ILA was the lead investigator for subjects gathered through the Ontario site of the BCFR and helped to draft the manuscript. EMJ was the lead investigator for subjects gathered through the Northern California site of the BCFR and helped to draft the manuscript. DEG contributed to study design, contributed to the analysis of rare variants in BRCA1 and BRCA2, gave advice on analysis of the MRN data, and helped to draft the manuscript. FL contributed to study design and data analysis, and helped to draft the manuscript. SVT was responsible for overall study design, contributed to data analysis, and helped to draft the manuscript. All authors read and approved the final manuscript.

## Supplementary Material

Additional file 1: Table S1 This spreadsheet contains anonymized coded observational data sufficient to perform most of the analyses of the MRN case-control mutation screening data described in this manuscript. Click here for file
